# Contingent negative variation: a biomarker of abnormal attention in functional movement disorders

**DOI:** 10.1111/ene.14189

**Published:** 2020-04-14

**Authors:** T. Teodoro, A. Koreki, A. M. Meppelink, S. Little, G. Nielsen, A. Macerollo, J. J. Ferreira, I. Pareés, A. Lang, M. J. Edwards

**Affiliations:** ^1^ Neurosciences Research Centre Molecular and Clinical Sciences Research Institute St George’s University of London London UK; ^2^ St George’s University Hospitals NHS Foundation Trust London UK; ^3^ Ashford and St Peter’s Hospital NHS Foundation Trust Chertsey UK; ^4^ Instituto de Medicina Molecular Faculdade de Medicina Universidade de Lisboa Lisboa Portugal; ^5^ Department of Neuropsychiatry National Hospital Organization Shimofusa Psychiatric Medical Center Chiba Japan; ^6^ Stichting Epilepsie Instellingen Nederland Zwolle The Netherlands; ^7^ Department of Clinical and Movement Neurosciences UCL Institute of Neurology, Queen Square London UK; ^8^ The Walton Centre Liverpool UK; ^9^ CNS‐Campus Neurológico Sénior Torres Vedras Portugal; ^10^ Neurology Department Hospital Ruber Internacional Madrid Spain; ^11^ Neurology Department Hospital Universitario Ramon y Cajal Madrid Spain; ^12^ Edmond J. Safra Program in Parkinson's Disease Morton and Gloria Shulman Movement Disorders Clinic Toronto Western Hospital UHN Division of Neurology University of Toronto Toronto Ontario Canada; ^13^ Krembil Brain Institute Toronto Ontario Canada

**Keywords:** biomarkers, functional neurological disorders, functional movement disorders, psychogenic disorders

## Abstract

**Background and purpose:**

Contingent negative variation (CNV) is a negative cortical wave that precedes a pre‐cued imperative stimulus requiring a quick motor response. It has been related to motor preparation and anticipatory attention. The aim was to ascertain whether the clinical improvement of functional movement disorders after physiotherapy would be associated with faster reaction times and modulation of CNV.

**Methods:**

Motor performance and CNV were analysed during a pre‐cued choice reaction time task with varying cue validity. Twenty‐one patients with functional movement disorders and 13 healthy controls at baseline were compared. Patients then underwent physiotherapy. At follow‐up after physiotherapy, patients were categorized as clinically improved (responders) or not improved (non‐responders) and retested.

**Results:**

At baseline, patients did not generate CNV, contrary to controls [mean amplitude (µV) at the end of preparation to move: patients −0.47 (95% CI −1.94, 1.00) versus controls −2.59 (95% CI −4.46, −0.72)]. Responders performed faster after physiotherapy [mean natural logarithm (ln) reaction time (RT) (ms): follow‐up 6.112 (95% CI 5.923, 6.301) versus baseline 6.206 (95% CI 6.019, 6.394), *P* = 0.010], contrary to non‐responders. Simultaneously, responders showed a recovery of CNV after physiotherapy [follow‐up −1.95 (95% CI −3.49, −0.41) versus baseline −0.19 (95% CI −1.73, 1.35), *P* < 0.001], contrary to non‐responders [follow‐up −0.32 (95% CI −1.79, 1.14) versus baseline −0.72 (95% CI −2.19, 0.75), *P* = 0.381].

**Conclusions:**

Clinical improvement of functional movement disorders after physiotherapy was associated with faster reaction times and normalization of CNV, which was absent at baseline. These findings suggest that CNV may constitute a useful neurophysiological biomarker related to abnormal attention in functional movement disorders.

## Introduction

One of the most characteristic clinical features of functional movement disorders (FMDs) is their alteration with attention: when attention is focused onto movement, movement is impaired; but with distraction, movement typically normalizes [1]. This phenomenon of abnormal explicit control of movement and normal implicit control underlies commonly used clinical and electrophysiological diagnostic tests of FMDs such as Hoover’s sign and entrainment and distractibility tests in functional tremor [2]. Attentional focus towards the mechanics of moving (i.e. monitoring the current state of the limb to be moved) forms a central feature of neurobiological models of FMD, whilst retraining attentional focus is a key part of specific physiotherapy‐based treatment programmes [1, 3, 4, 5]

There is some evidence of a pathophysiological role for explicitly directed attention in FMD [1, 6]. Therefore, experimental techniques that directly probe explicitly directed attention could help to identify potential biomarkers for FMDs [6]. A useful biomarker would be abnormal in people with FMD when they were symptomatic and would normalize if improvement of symptoms occurred.

The usefulness of a simple pre‐cued reaction time (RT) task, based on the classic Posner paradigm, as a diagnostic biomarker for FMD was previously explored [1, 6]. In this paradigm, a pre‐cue predicts with varying probability which movement will be required (a button press with the right or left hand) following an upcoming ‘go’ cue. In an initial behavioural study, it was shown that people with FMD, in contrast to healthy controls, did not improve their RT in response to a pre‐cue that reliably predicted the type of movement they were required to make [1]. In a subsequent study, this behavioural effect was replicated and it was shown that the normal desynchronization of beta power that can be detected by electroencephalography (EEG) prior to cued movement was not present in people with FMD performing this task [6]. A non‐significant trend for recovery of this beta desynchronization was found in people with FMD who had improved clinically following specific physiotherapy treatment [6]. This suggested that excessive synchronization of brain activity on the beta band could constitute a biomarker for abnormal movement preparation in FMD [6].

In this study, the utility of a different potential biomarker was explored: the contingent negative variation (CNV). CNV is a slow negative cortical wave that develops following a pre‐cue which signals that, within a few seconds, an imperative stimulus will arrive, requiring a quick motor response [7, 8]. CNV is regarded as an ‘expectancy wave’, reflecting anticipatory attention and motor preparation to react to the forthcoming cue [8, 9].

In people with FMD, it was predicted that excessive attention onto the current state of the limb to be moved and away from the movement’s goal would be associated with an insufficient deployment of anticipatory attention and abnormal motor preparation. This would translate into slower RTs to the imperative cues (thus replicating the findings of previous research) [1] and a reduction in CNV amplitudes. In line with our expectations, a previous study of six patients with functional weakness found a reduction in CNV amplitude which was not evident in controls feigning weakness [10], and a pre‐movement potential before self‐paced voluntary movement has been reported to be absent in people with functional jerks [11].

Therefore, in this study, it was hypothesized that (i) CNV amplitude would be reduced at baseline in comparison with healthy controls; (ii) clinical improvement after physiotherapy would be associated with faster RT and recovery of CNV.

## Methods

### Participants, experimental task and EEG recording

A case–control study was performed comparing patients with FMD and healthy volunteers. Patients with FMD were recruited from a pool of patients being enrolled in a randomized feasibility study comparing specialized with standard physiotherapy for FMD [5]. A detailed description of the specialized physiotherapy programme is given in Appendix S1. These subjects were ≥ 18 years old and had a clinically established diagnosis of FMD according to the Fahn–Williams criteria [12]. All patients attended a consultation with the study neurologist (MJE). Additional inclusion criteria were a symptom duration of at least 6 months, functional motor symptoms causing significant disability, a completed diagnostic investigation and acceptance of the diagnosis of FMD. Relevant exclusion criteria were the presence of pain or fatigue as the primary cause of disability, prominent dissociative seizures, clinically significant depression or anxiety and a high level of disability preventing participation in an outpatient environment [5]. FMD participants were tested before starting physiotherapy (baseline) and at least 2 weeks after completing treatment (follow‐up) (Table 1). Matched healthy controls were assessed only once [6]. Phenomenology at baseline was characterized on the basis of a video rating by three neurologists, as described elsewhere (Table 1) [13].

**Table 1:** Functional movement disorder patients at baseline versus follow‐up: demographics and response to treatment

Groups	FMD responders	FMD non‐responders
*N* total	10	11
Sex (males/females)	2/8	2/9^a^, *
Age, years (median, IQR)	43 (30–45)	41 (36–53)^a^, *
Phenomenology^b^		
Gait impairment	7	7
Motor slowness	0	1
Incoordination	1	1
Upper limb tremor	2	4
Head tremor	0	2
Trunk tremor	1	1
Axial myoclonus	1	1
Functional dystonia	1	1
Upper limb involvement (any)	3	5
Bilateral involvement	6	7
Right‐sided involvement	0	3
Left‐sided involvement	3	1
Number of patients who received specialized physiotherapy	8/10	1/11^a^, *
SF‐36 (median, IQR)		
Baseline	30 (20–50)	25 (10–30)
Follow‐up at 6 months	60 (35–80)^c^, *	15 (5–40)^c^, *
S‐FMDRS (median, IQR)		
Baseline	15 (9–21)	14 (12–18)
Follow‐up at 6 months	5 (2–13)^c^, *	24 (16–33)^c^, *

SF‐36 baseline versus follow‐up (Wilcoxon sign‐rank test): for responders *P* = 0.021; for non‐responders *P* = 0.433.

S‐FMDRS baseline versus follow‐up (Wilcoxon sign‐rank test): for responders *P* = 0.044; for non‐responders *P* = 0.074.

FMD, functional movement disorder; IQR, interquartile range; SF‐36, Short‐Form Health Survey; S‐FMDRS, Simplified Functional Movement Disorders Rating Scale.

^a^ Responders versus non‐responders.

^b^ Based on baseline video rating by three neurologists [13].

^c^ Baseline versus follow‐up.

* *P* < 0.05; **P ≥ 0.05.

Assessment of clinical improvement after physiotherapy was based on Clinical Global Impression (CGI), the Physical Function domain of the Short‐Form Health Survey (SF‐36) (version 1) and the Simplified Functional Movement Disorders Rating Scale (S‐FMDRS) [5, 13, 14]. Patients with FMD were dichotomized as responders or non‐responders to physiotherapy, based on their self‐rated CGI [5]. Responders self‐rated themselves as improved or much improved after physiotherapy. Non‐responders self‐rated as unchanged, worse or much worse. Our study was nested within a randomized feasibility trial that used the same criteria for collapsing the CGI [5]. The Physical Function domain of the SF‐36 questionnaire focuses on motor function, inquiring about limitations on 10 mobility activities [14]. Finally, the S‐FMDRS is a simplified version of the Functional Movement Disorders Rating Scale and has shown good inter‐rater reliability and sensitivity to change [13]. The raters of S‐FMDRS were blinded for time‐point of assessment (before versus after treatment), as reported elsewhere [13].

Our behavioural experiment consisted of a Posner‐type pre‐cued choice RT task with varying cue validity [1, 6, 15], including (i) a highly predictable condition, where preparation cues accurately predicted go cues in 95% of the trials (95% congruence); (ii) an unpredictable condition, where preparation cues accurately predicted go cues in only 50% of the trials (50% congruence). Participants were instructed to press the key corresponding to the go cue as quickly as possible (either the left Ctrl key with left index finger or right Ctrl key with right index finger). A flowchart with the trial structure was included in a previous publication [6].

Response time in milliseconds (ms) was calculated for each trial. Trials where the preparation cue accurately predicted the go cue (congruent) were separated from those where the prediction was incorrect (incongruent). RTs were separately averaged across trials for congruent and incongruent trials in each of the two conditions.

Continuous EEG was recorded using a 32‐channel ANT‐EEG® (ANT Neuro, Hengelo, Netherlands) system conforming to the 5% electrode system. Our reference was an average of all electrodes. Trials with prominent artefacts and trials where participants pressed the wrong key or did not press any key were excluded.

A more detailed description of the participants, experimental task and EEG recording can be found elsewhere [6].

### Pre‐processing

Statistical Parametric Mapping (12b, The Wellcome Centre for Human Neuroimaging, UCL Queen Square Institute of Neurology, London, UK) and MATLAB® (MathWorks, Natick, MA, USA) were used for data processing. Data were downsampled from 2048 to 250 Hz and epoched to frames from −1 to +4 s relative to the onset of the preparation cue. The interval preceding the preparation cue was selected as baseline and baseline‐corrected the epoched frames. Finally, data were averaged over trials for each participant and extracted data from the Cz electrode (amplitude, µV), which is considered to record CNV with greatest amplitude [16]. The midline location of Cz also facilitated combining data from right and left key presses. This maximized the statistical power to compare subgroups of patients with FMD who improved and did not improve after physiotherapy (see below).

Pre‐processing resulted in four datasets of Cz amplitude as a function of time: (a) 95% trial, right key press (right index finger); (b) 95% trial, left press (left index finger); (c) 50% trial, right press; (d) 50% trial, left press.

### Statistical analysis

Statistical analysis was performed using Stata® (version 13.1, College Station, TX, USA,). Continuous variables were expressed as mean (and standard deviation) if normally distributed or median (and interquartile range) if not normally distributed. Categorical variables were expressed as frequencies and proportions. The normality assumption was assessed by visually inspecting the distribution of the continuous variable and confirmed by Kolmogorov–Smirnov testing.

Reaction times (RTs) were non‐normally distributed and were therefore transformed into their natural logarithms (ln), in order to fulfil the normality assumption and thus be able to fit a multilevel mixed effect linear model.

Participants could pre‐plan the forthcoming key press in the interval between the appearance of preparation and go cues (interval duration 1950 ms). CNV amplitude (µV) at the moment of maximum preparation was analysed by restricting our analysis to the last 12 ms preceding the go cue (averaging data from three data points).

Our outcome measures were RT (ms) and CNV amplitude (µV) at the end of preparation to move. Mixed effects multilevel linear modelling allowed the dependence in the data caused by repeated measurements within‐subjects to be taken into account. The following models were fitted.

- A baseline comparison was made of patients with FMD and healthy controls.
  - Behavioural results (RTs) for baseline comparison were presented in our previous paper focusing on beta oscillations (see summary below) [6].
  - For CNV amplitude, the effects of group, predictability and hand, their interactions and an individual level random effect were included.
- A comparison was carried out of FMD responders and non‐responders to physiotherapy, before and after this intervention.
  - For RT, our analysis was restricted to trials with congruent preparation and go cues, as those were the ones thought to reflect motor preparation. A model was fitted including the effects of time‐point (baseline versus follow‐up), response (responder versus non‐responder) and predictability (95% vs. 50%), their respective interactions and an individual level random effects factor.
  - For CNV amplitude, the effects of group, predictability and hand, their interactions and an individual level random effect were included.

Finally, the relationship between changes in CNV and changes in RT at follow‐up was investigated. The grand average of end‐of‐preparation CNV (µV) and RT (ms) at baseline and at follow‐up was calculated, for each participant. The baseline averages were then subtracted from the follow‐up averages for both parameters. It was planned to regress the average change of RT against the average change of end‐of‐preparation CNV.

Statistical significance was predefined as *P* < 0.05.

## Ethics

This study was approved by the local ethics committee. Participants gave their informed written consent to take part in the studies.

## Results

### Clinical and demographic characteristics

Twenty‐one patients with FMD and 13 healthy controls were recruited and a baseline assessment was performed. Nine patients with FMD were randomized to undergo specialized physiotherapy and another 12 to receive standard physiotherapy. Groups at baseline were well matched for age, sex and proportion of left‐handed participants (reported elsewhere [6]).

Patients with FMD were evaluated after a mean period of 4.7 weeks (SD 1.7) after treatment. Ten patients with FMD were classified as responders and 11 as non‐responders, in accordance with their self‐rated CGI. FMD responders, contrary to non‐responders, showed an increase in SF‐36 and a decrease in S‐FMDRS at follow‐up (Table 1). The age and sex proportions were similar in both groups. Eight out of 10 responders and one out of 11 non‐responders had been randomized to receive specialized physiotherapy, whilst the others underwent standard physiotherapy [5].

### Functional movement disorder patients at baseline versus healthy controls

#### Behavioural results

**Figure 1:**
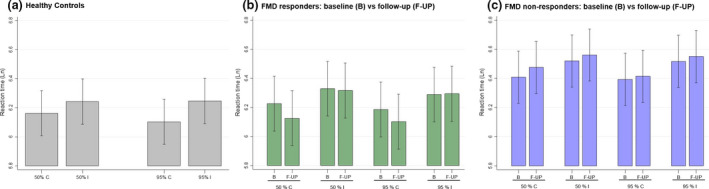
Natural logarithm of reaction time per group, predictability and cue congruence. 50%, 50% congruence blocks (including congruent cues in 50% trials); 95%, 95% congruence blocks; C, trials with congruent cues; I, trials with incongruent cues. [Colour figure can be viewed at wileyonlinelibrary.com]

For RT, it has been previously reported elsewhere [6] that healthy controls performed faster in trials with predictive pre‐cues compared with trials with non‐predictive pre‐cues [mean ln(RT) predictive pre‐cues 6.104 (95% CI 5.947, 6.261) versus non‐predictive pre‐cues 6.162 (95% CI 6.006, 6.319), *P* = 0.032] (Fig. 1). In contrast, in patients with FMD, RTs were similar in predictive and non‐predictive pre‐cues [mean ln(RT) predictive pre‐cues 6.287 (95% CI 6.166, 6.408) versus non‐predictive pre‐cues 6.314 (95% CI 6.194, 6.435), *P* = 0.206].

#### End‐of‐preparation CNV

**Figure 2:**
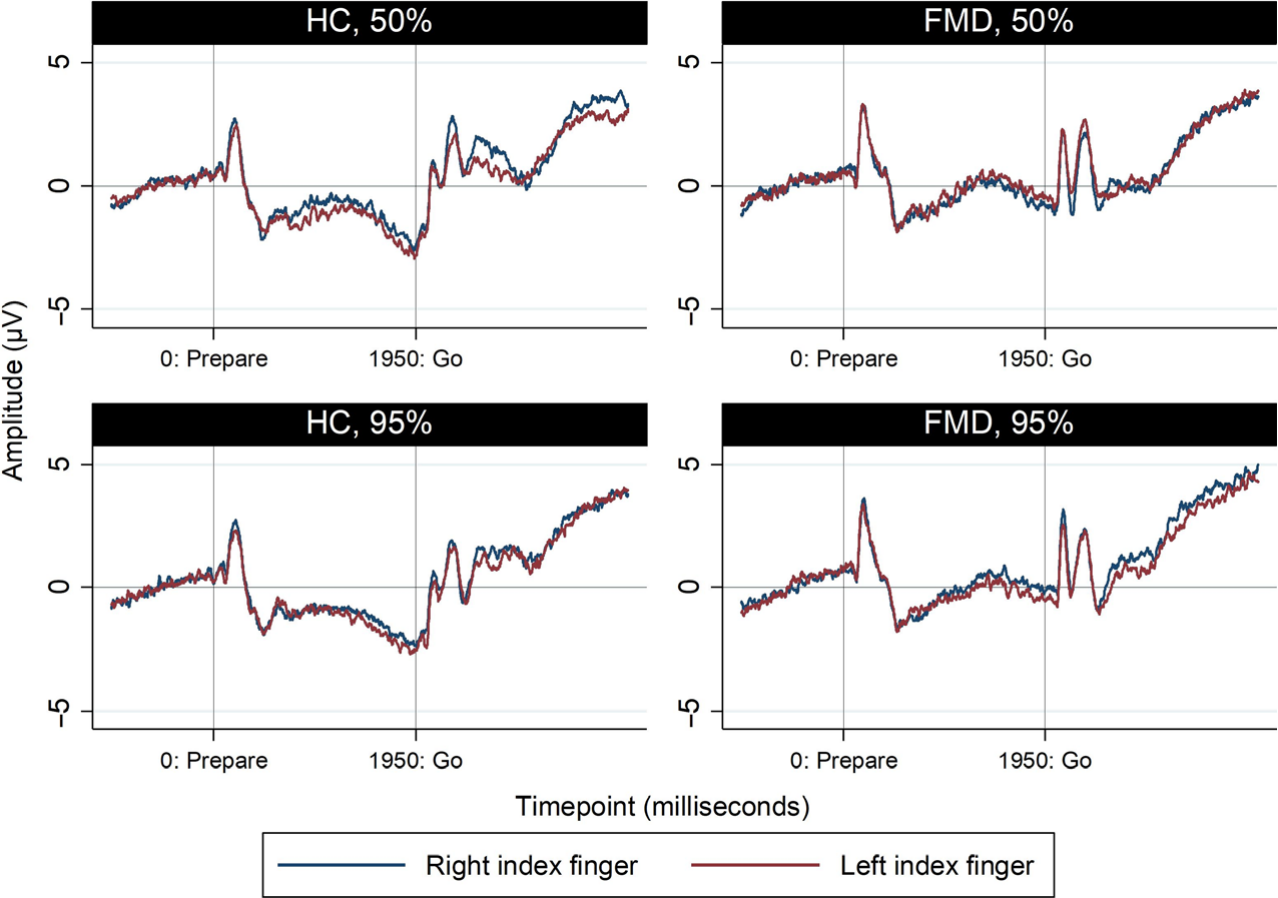
End‐of‐preparation CNV: FMD patients at baseline versus healthy controls. [Colour figure can be viewed at wileyonlinelibrary.com]

A significant effect for group (*P* = 0.050) was found but not for predictability (*P* = 0.484), hand (*P* = 0.496) or the interactions group × predictability (*P* = 0.459), group × hand (*P* = 0.245), predictability × hand (*P* = 0.923) and group × predictability × hand (*P* = 0.361) (Fig. 2, Table S4).

After eliminating all non‐significant factors from our model, the *P* value for the pairwise comparison between FMD and healthy controls was 0.081 [mean FMD −0.47 (95% CI −1.94, 1.00) versus healthy controls −2.59 (95% CI −4.46, −0.72)]. Importantly, patients with FMD failed to generate the negative wave that defines CNV (*P* = 0.532 for rejecting the null hypothesis of CNV amplitude being zero), contrary to healthy controls (*P* = 0.007).

### Functional movement disorder patients at follow‐up versus baseline

#### Behavioural results

In our predefined model of normalized RT, the only significant effect was for the interaction response × time‐point (*P* = 0.012). None of the other terms was significant, including response (*P* = 0.184), time‐point (*P* = 0.140), predictability (*P* = 0.755), response × predictability (*P* = 0.691), time‐point × predictability (*P* = 0.466) and response × time‐point × predictability (*P* = 0.498). Responders at follow‐up were unable to take advantage of predictive conditions (95% congruence) to perform faster, compared with non‐predictive conditions (50% congruence) (*P* = 0.643 for the corresponding pairwise comparison). This specific finding is similar to what has been described elsewhere for patients with FMD at baseline [6].

In order to dissect the significant interaction between response × time‐point, a pairwise comparison analysis was then performed in a model only including response, time‐point and their interaction. In accordance with our predictions, responders performed faster at follow‐up than at baseline [mean ln(RT) at follow‐up 6.112 (95% CI 5.923, 6.301) versus baseline 6.206 (95% CI 6.019, 6.394), *P* = 0.010] whilst non‐responders’ performance was similar [mean ln(RT) at follow‐up 6.444 (95% CI 6.265, 6.623) versus baseline 6.401 (95% CI 6.222, 6.579), *P* = 0.185].

See Table S1 for non‐normalized RT, Fig. 1 and Table S2 for the corresponding natural logarithms and Table S3 for the accuracy results.

#### End‐of‐preparation CNV

**Figure 3:**
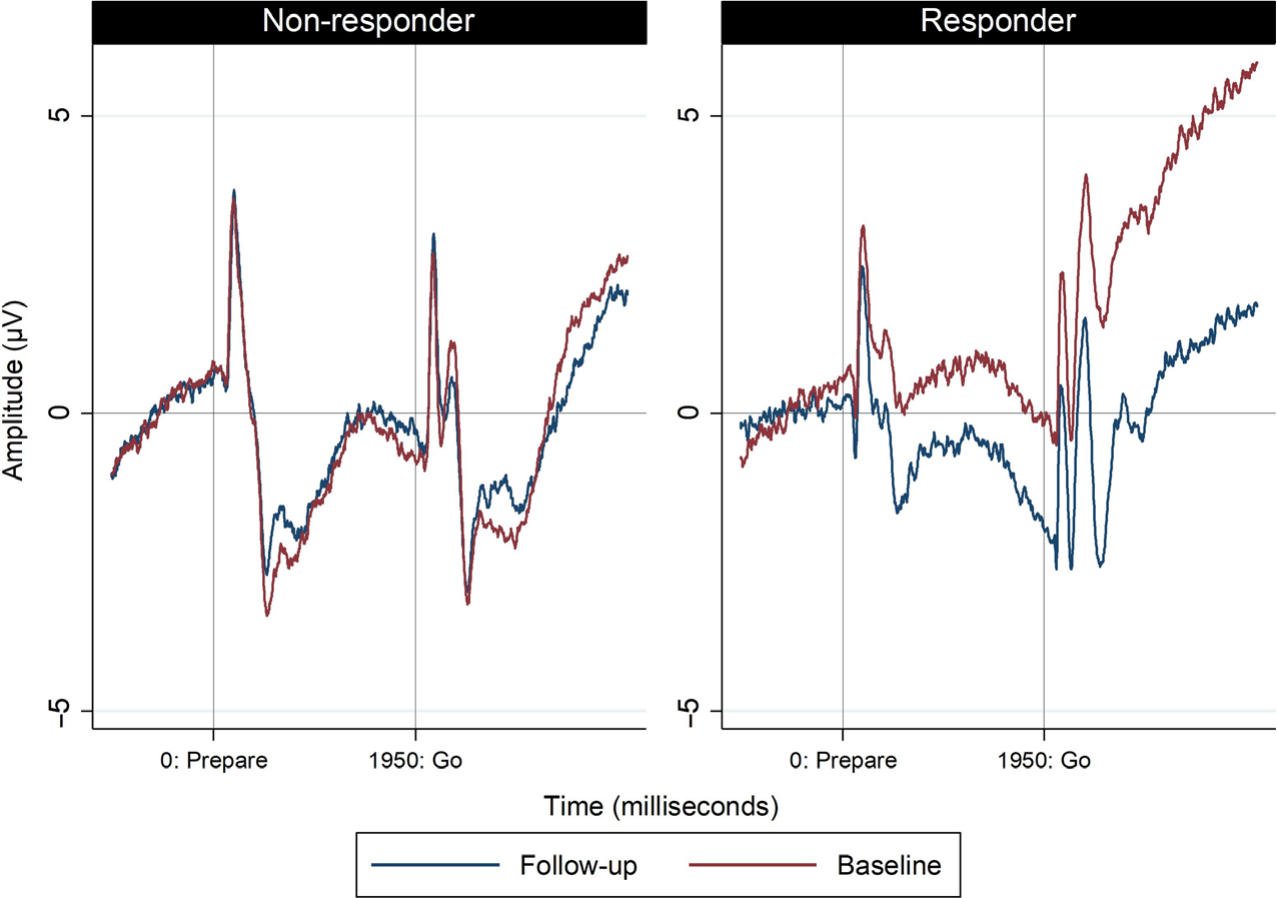
End‐of‐preparation CNV: FMD responders and non‐responders at baseline versus follow‐up. [Colour figure can be viewed at wileyonlinelibrary.com]

In our predefined model, the effects of response (*P* = 0.626) and time‐point (*P* = 0.381) were non‐significant but their interaction was significant (*P* = 0.001) (Fig. 3, Table S4). In order to clarify this interaction, a pairwise comparison analysis was performed. After physiotherapy, the power at the end of preparation to move became more negative in responders [mean, follow‐up −1.95 (95% CI −3.49, −0.41) versus baseline −0.19 (95% CI −1.73, 1.35), *P* < 0.001] but not in non‐responders [mean, follow‐up −0.32 (95% CI −1.79, 1.14) versus baseline −0.72 (95% CI −2.19, 0.75), *P* = 0.381]. Notably, only responders at follow‐up generated a negative wave at the end of preparation to move [mean −1.95 (95% CI −3.49, −0.41), *P* = 0.013].

##### Relationship between changes in RT and in CNV at follow‐up

In responders, RT became −41 ms (SD 31) faster at follow‐up, whilst the end‐of‐preparation CNV became −1.97 (SD 2.12) more negative at follow‐up. In contrast, in non‐responders, RT became 12 ms (SD 159) slower and the end‐of‐preparation CNV 0.40 (SD 4.56) more positive at follow‐up.

**Figure 4:**
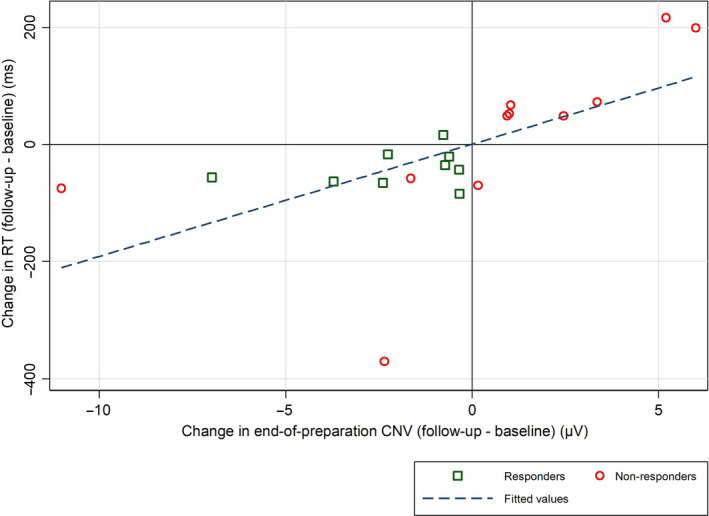
Relationship between changes in RT and in CNV at follow‐up. [Colour figure can be viewed at wileyonlinelibrary.com]

In the linear regression of changes in RT against changes in end‐of‐preparation CNV, the RT became −19 ms faster for each −1 µV increase in CNV negativity (*P* = 0.004) (Fig. 4).

## Discussion

Here it is reported that CNV is abnormal in people with FMD and that clinical improvement that occurred following treatment is associated with its normalization. In contrast, people with FMD who did not experience clinical improvement with treatment continued to demonstrate abnormal CNV at follow‐up assessment.

### Suppression of CNV and abnormal motor preparation in FMD

It has been previously observed that people with FMD are unable to take advantage of highly predictable conditions to prepare for the forthcoming movement and improve performance (i.e. generate faster RTs) [1, 6]. This finding is in accordance with their difficulty in performing movements in an explicit context (e.g. to command during a physical examination), but retained ability for normal movement to occur when happening in an automatic or implicit manner. It has been previously proposed that this reflects a misdirection of attention towards the mechanics of a movement and away from its goal, in line with neurobiological accounts of FMD [1, 17]. It has recently been demonstrated that this behavioural phenomenon is associated with persistent beta synchronization during motor preparation, which showed a non‐significant trend towards recovery of normal beta suppression prior to movement, following clinical improvement after treatment [6].

Contingent negative variation is related to anticipatory attention and motor preparation [8, 9]. Therefore, the suppression of CNV observed in our patients at baseline probably reflects abnormalities in motor preparation and attention, in keeping with the mechanism hypothesized in the Introduction [1, 17].

Only one previous study reported suppression of CNV in FMD, in a group of six patients with functional weakness. Suppression of CNV was not observed in a group of 24 participants feigning paralysis, despite similar motor performances, or in a group of 12 healthy subjects [10].

In addition, FMD patients were highly accurate in their performance (95.3% vs. 98.8% in controls) which it is believed is evidence against feigning as an explanation for their lack of CNV (Table S3).

### Functional improvement and recovery of CNV

It was found that clinical improvement in responders was associated not only with faster RT but also with a recovery of CNV after treatment. The same was not observed in non‐responders, ruling out confounding by a simple retesting effect. Physiotherapy for FMD is based on movement retraining with the aim of restoring normal movement by redirecting the focus of motor attention towards the movement goal and away from movement mechanics [4, 5]. CNV recovery at follow‐up could therefore plausibly reflect a successful retraining of movement, with a refocusing of motor attention towards the movement goal. To our knowledge, only one previous study has reported change in a neurobiological marker of nervous system dysfunction following successful treatment [18]. Vuilleumier *et al*. observed a decrease in thalamic and basal ganglia single‐photon emission computed tomography activation in response to contralateral limb vibration in seven patients with unilateral functional motor symptoms, which normalized after symptom improvement at follow‐up [18].

### Cues to interpret previous findings on Bereitschaftspotentials

Our results may help explain rather unusual results from assessment of Bereitschaftspotentials (BPs, pre‐movement potentials recorded prior to self‐paced movement) in people with functional myoclonus [11]. In these patients, functional jerks are associated with the expected presence of a BP, but, intriguingly, voluntarily mimicked jerks are not associated with a BP. Taken together, these results point to a general problem in voluntary movement (self‐paced or externally paced), which is reflected in abnormalities of cortical potentials associated with movement preparation.

### Relevance of symptom distribution

A crucial facet of the data presented here is that CNV relating to movement preparation for right or left arm movement was recorded, but many of the patients did not have symptoms in their arms or, in some patients, only one arm was affected. Despite this, there was no systematic difference in our findings between those with or without clinical involvement of the upper limbs. This fits with our clinical experience that it is very common for functional motor signs to be triggered through the act of physical examination, even in patients who do not complain of specific symptoms in the limb being examined. Indeed this phenomenon is commonly seen in people with non‐motor functional symptoms, e.g. chronic pain, functional sensory loss, chronic fatigue. In such patients, examination of power commonly reveals give‐way patterns of weakness, a positive Hoover’s sign, or flurries of jerks and tremors. This is in accordance with the common co‐occurrence of functional symptoms in different domains (motor, exteroception, interoception) and with neurobiological accounts of functional neurological disorders which make no separation between the mechanism of functional symptoms that occur in different domains. This is important information for the potential use of CNV as a neurophysiological diagnostic biomarker, as it does not require people to have symptoms in the limbs being studied, and it also may be of use in those with non‐motor functional symptoms. This requires further study, but could indicate a more general utility of CNV as a biomarker related to abnormal attention in functional neurological disorder.

## Limitations

There are several limitations to our study. It was decided to use data from the lead Cz because this is previously reported to provide CNV with the largest amplitude. Our main interest here was studying FMD responders and non‐responders, which restricted our sample size. Therefore, it was decided to prioritize testing for differences in CNV amplitudes over investigating CNV lateralization, which is obviously not possible with Cz.

Patients were dichotomized into responders and non‐responders based on one self‐rated outcome measure (CGI). However, changes in SF‐36 (self‐reported quality of life measure) and S‐FMDRS (video rating blinded for time‐point [13]) after treatment also supported our criteria for collapsing groups over CGI (Table S1–S4). It is acknowledged that abnormalities in CNV (using different paradigms from ours) have been reported in other disorders. For example, CNV attenuation has been described in Parkinson’s disease [19], schizophrenia [20] and attention‐deficit hyperactivity disorder [21], and an enhancement was observed in Gilles de la Tourette syndrome [22]. It would be useful for future studies to include movement disorder disease control groups to understand the nature of the overlap between CNV abnormalities in people with FMD and those with other disorders.

Experiments with long intervals between preparation and go cues have described an early and late component of CNV. Notwithstanding significant controversy, late CNV was proposed to be more closely related with the BP. Although the rules for decomposing CNV into its early and late components are not ‘set in stone’, foreperiods of at least 3 s duration are often used. Therefore, it is considered that our interval was too short to allow a precise separation of these components.

Responders’ RTs in congruent trials overall became faster at follow‐up, contrary to what was observed in non‐responders. However, there was a persistence of some behavioural abnormalities, with patients with FMD remaining unable to take advantage from predictive pre‐cues to perform even faster (contrary to healthy controls, as reported elsewhere [6]).

Contingent negative variation abnormalities were previously described in other movement disorders, including Parkinson’s disease [19], writer’s cramp [23], cervical dystonia [24] and Huntington disease [25]. Therefore, abnormal CNV is not specific to patients with FMD, which limits its utility for the differential diagnosis with other movement disorders.

In conclusion, a recovery of CNV in the context of a clinical and behavioural improvement after physiotherapy is described. These findings suggest that CNV is a potential candidate biomarker for treatment response in FMD, and indeed may have utility outside the setting of those with FMDs and be useful in functional neurological disorder in general.

## Authors' roles

(i) Research project: conception, MJE, AL, TT, IP, SL, AMM; organization, AMM, IP, TT, GN, SL, MJE; execution, AMM, IP, TT, GN, AM, IP. (ii) Statistical analysis: design, TT, AK; execution, TT, AK; review and critique, TT, AK, MJE, SL, IP, JJF. (iii) Manuscript: writing of the first draft, TT; review and critique, AK, AMM, SL, GN, AM, JJF, IP, AL, MJE.

## Disclosure of conflicts of interest

Tiago Teodoro, Akihiro Koreki, Anne Marthe Meppelink, Simon Little, Glenn Nielsen, Antonella Macerollo and Mark J Edwards: no disclosures. Joaquim J Ferreira: stock ownership in medically related fields – none; intellectual property rights – none; consultancies – GlaxoSmithKline, Novartis, TEVA, Lundbeck, Solvay, Abbvie, BIAL, Merck‐Serono, Merz, Ipsen, Biogen, Sunovion Pharmaceuticals, Zambon; expert testimony – BIAL, Novartis; advisory boards – BIAL, Sunovion Pharmaceuticals; employment – Faculdade de Medicina de Lisboa, CNS – Campus Neurológico Sénior; partnerships – none; contracts – none; honoraria – none; royalties – none; grants – GlaxoSmithKline, Grunenthal, Fundação MSD (Portugal), TEVA, MSD, Allergan, Ipsen, Novartis, Medtronic; other – none. Isabel Pareés: has received honoraria and/or travel expenses from Allergan, Abbvie, Alter, TEVA and Italfarmaco for attending and/or speaking at meetings. Anthony Lang: stock ownership in medically related fields – none; consultancies – Abbvie, Acorda, Biogen, Janssen, Jazz Pharma, Sun Pharma, Kallyope, Merck, Paladin, Theravance and Corticobasal Degeneration Solutions; advisory boards – Jazz Pharma, PhotoPharmics; partnerships – none; honoraria – Sun Pharma, Medichem, Medtronic, AbbVie and Sunovion; grants – Brain Canada, Canadian Institutes of Health Research, Corticobasal Degeneration Solutions, Edmond J Safra Philanthropic Foundation, Michael J. Fox Foundation, the Ontario Brain Institute, National Parkinson Foundation, Parkinson Society Canada, and W. Garfield Weston Foundation; intellectual property rights – none; expert testimony – none; employment –University Health Network, University of Toronto; contracts – none; royalties – Elsevier, Saunders, Wiley‐Blackwell, Johns Hopkins Press and Cambridge University Press; other – none.

## Supporting information


**Table S1.** Reaction time (non‐transformed) per group and time‐point
**Table S2** **.** Reaction time (natural logarithm) per group and time‐point
**Table S** **3.** Accuracy per group and time‐point
**Table S** **3.** CNV at end of preparation to move

**Appendix S1.** Specialist physiotherapy for functional motor symptoms
