# Supplementary material for: Contingent negative variation: a biomarker of abnormal attention in functional movement disorders
Source: Eur J Neurol. 2020 Apr 14;27(6):985–94. doi: 10.1111/ene.14189 (PMC7982797; doi:10.1111/ene.14189)
Supplement: Supplementary file 1 — Table S1. Reaction time (non‐transformed) per group and time‐point Table S2 . Reaction time (natural logarithm) per group and time‐point Table S 3. Accuracy per group and time‐point Table S 3. CNV at end of preparation to move [file ENE-27-985-s001.docx]

**Supplementary Table I: Reaction time (non-transformed) per group and time-point**

| Group | |  | Reaction time (ms) (Median [IQR])  (only trials with congruent cues) | |
| --- | --- | --- | --- | --- |
|  |  | | Baseline | Follow-up |
| **HC** | | | 469 [406-536] |  |
| **FMD** | | | 513 [426-599] |  |
| **FMD “non-responders”** | | | 541 [458-728] | 581 [500-704] |
| **FMD “responders”** | | | 486 [419-543] | 431 [383-472] |

**Supplementary Table II: Reaction time (natural logarithm) per group and time-point**

| Group | |  | Natural logarithm of reaction time (Mean [SD])  (only trials with congruent cues) | |
| --- | --- | --- | --- | --- |
|  |  | | Baseline | Follow-up |
| **HC** | | | 6.133 [0.044] |  |
| **FMD** | | | 6.308 [0.055] |  |
| **FMD “non-responders”** | | | 6.401 [0.392] | 6.444 [0.322] |
| **FMD “responders”** | | | 6.206 [0.281] | 6.042 [0.148] |

**Supplementary Table III: Accuracy per group and time-point**

| Group | |  | Accuracy (% of Failed Trials) [Median (IQR)] | |
| --- | --- | --- | --- | --- |
|  |  | | Baseline | Follow-up |
| **HC** | | | 1.25 [1.25-2.25] |  |
| **FMD** | | | 4.75 [1.5-9.5] |  |
| **FMD “responders”** | | | 3.25 [1.25-12] | 2 [1-3] |
| **FMD “non-responders”** | | | 7 [1.5-9.5] | 4.5 [2.25-11.25] |

FMD at baseline vs HC (p 0.022); FMD “responders” vs “non-responders” at baseline: p 0.526; FMD “responders” vs “non-responders” at follow-up: p 0.110; FMD “non-responders” baseline vs follow-up: p 0.625; FMD “responders” baseline vs follow-up: p 0.033 (uncorrected p-values)

**Supplementary Table IV: CNV at end of preparation to move**

| Group | |  | "End of preparation" CNV (µV) (Mean [SD]) | |
| --- | --- | --- | --- | --- |
|  |  | | Baseline | Follow-up |
| **HC** | | | -2.59 [3.44]* |  |
| **FMD** | | | -0.47 [3.68] |  |
| **FMD “non-responders”** | | | -0.72 [4.57] | -0.32 [2.42] |
| **FMD “responders”** | | | -0.19 [2.43 ] | -1.95 [2.47]* |

(^*^) - Different from zero (p < 0.05)
