# Supplementary material for: Contingent negative variation: a biomarker of abnormal attention in functional movement disorders
Source: Eur J Neurol. 2020 Apr 14;27(6):985–94. doi: 10.1111/ene.14189 (PMC7982797; doi:10.1111/ene.14189)
Supplement: Supplementary file 2 — Appendix S1. Specialist physiotherapy for functional motor symptoms [file ENE-27-985-s002.docx]

**Appendix S1: Specialist Physiotherapy for Functional Motor Symptoms**

Description of treatment following the TIDieR Checklist^1^

The following table summarises the specialist physiotherapy intervention completed by subjects in this study. We have described our specific treatment approach in more detail elsewhere.^2,3^

| **1. Name** | **Provide the name or a phrase that describes the intervention.** |
| --- | --- |
|  | Specialist physiotherapy for functional motor symptoms (FMS) |
|  |  |
| **2. Why** | **Describe the rationale, theory, or goal of the elements essential to the intervention.** |
|  | The rationale for the specialist physiotherapy treatment is primarily based on a particular aetiological model for FMS that highlights attention and expectation as key mechanisms that drive symptoms.^4^  Functional motor symptoms require the patient’s attention, at a level without voluntary control, to be directed towards their body in order to manifest. When the patient’s attention is distracted, the movement disorder disappears or dampens.  The patient has an expectation, at a level without voluntary control, that their movement will be abnormal; this expectation is associated with a particular illness belief (e.g. my legs are paralysed). Expectations of abnormal movement influence motor output with symptoms arising as a ‘habit’ that the nervous system has got in to.  The intervention addresses attention-related movement problems by retraining activity (movement) while redirecting the patient’s focus of motor attention. Altered expectations and illness beliefs are addressed through education, demonstrating to the patient that they can move normally and helping the patient to develop strategies that normalises their movement during every day activities.  The main components of the intervention are:   1. A joint consultation with the neurologist and physiotherapist where diagnostic information is reviewed and the aims of the programme discussed. The aims of treatment were described as retraining movement and learning how to manage symptoms in the longer term. 2. Education about FMS. Education is based on the aetiological model described above, the patient and physiotherapist collaboratively devise a symptom formulation taking into account triggering events, comorbidity, psychological factors, self-focused attention ad unhelpful reinforcement of symptomatic movement patterns.^5^ 3. Movement retraining. Movement retraining aimed to restore normal movement during problematic activities through task practice while redirecting the focus of motor attention. 4. Developing a symptom management plan. During treatment the patient and physiotherapist make notes in a workbook, this information is summarised to form a symptom management plan at the end of treatment. |
|  |  |
| **3. What: Materials** | **Describe any physical or informational materials used in the intervention, including those provided to participants or used in intervention delivery or in training of intervention providers. Provide information on where the materials can be accessed (such as online appendix, URL).** |
|  | Patient Workbook: Patients are given a workbook which is completed by both the patient and the physiotherapist during treatment. Key sections of the workbook are:   1. Understanding the diagnosis 2. Neuroanatomy and physiology 3. Pages for patients to reflection on sessions 4. Movement retraining 5. Understanding problems associated with FMS (pain, fatigue and memory problems) 6. Self-management plan. |
|  |  |
| **4. What: Procedures** | **Describe each of the procedures, activities and/or processes used in the intervention, including any enabling or support activities.** |
|  | Neurology: The diagnosis of FMS is made and explained to the patient following a standardized explanation that emphasises symptom mechanisms.  Physiotherapy – Education: Patients receive a standardised explanation of FMS using the workbook as a guide. This is followed by an individualised formulation, where the patient and physiotherapist collaboratively devise a theoretical explanation for how the person came to develop FMS, using a symptom model. The formulation seeks to determine relevant risk factors, triggers, initial symptoms, examples of attention affecting movement, adaptive coping strategies, secondary changes, and social factors. Education includes information about some common problems associated with FMS (pain, fatigue, and memory/concentration).  Physiotherapy – Movement Retraining**:** Movement retraining generally follows a sequential motor learning approach, building up desired movement patterns starting from elementary, symptom free components of movement. Movement retraining focuses in tasks such as sit to stand/stand to sit, walking, using stairs, drinking from a cup, etc. Movement retraining may involve standard physiotherapy adjuncts such as electrical muscle stimulation and treadmill training.  Physiotherapy – Personal Reflections: At the end of each physiotherapy session, the patient is encouraged to write a reflection in their workbook.  Physiotherapy – Self-Management: To conclude treatment, a personalised self-management plan is developed, which usually includes: (i) a summary of useful strategies that help to normalise movement; (ii) activity plans to address boom and bust patterns and how to progress activity; (iii) future goals; and (iv) what to do on difficult days and during periods of symptom exacerbation. |
|  |  |
| **5. Who provided** | **For each category of intervention provider, describe their expertise, background and any specific training given.** |
|  | Neurologist: Consultant neurologist with experience in functional movement disorders.  Physiotherapist: Senior physiotherapist specialised in neurology, with experience and additional training in functional movement disorders. |
|  |  |
| **6. How** | **Describe the modes of delivery (such as face to face or by some other mechanism, such as internet or telephone) of the intervention and whether it was provided individual or in a group.** |
|  | Each session is conducted face to face and individually (there are no group treatment sessions). |
|  |  |
| **7. Where** | **Describe the type(s) of location(s) where the intervention occurred, including any necessary infrastructure or relevant features.** |
|  | Physiotherapy sessions are held in a physiotherapy gym/clinic with space suitable for movement and gait retraining and space suitable for education and writing in the intervention workbook. Additional equipment includes a full-length mirror and treadmill. |
|  |  |
| **8. When and how much** | **Describe the number of times the intervention was delivered an over what period of time including the number of sessions, their schedule and their duration, intensity or dose.** |
|  | Patients are admitted to a day hospital for 5 consecutive days. They receive 8 sessions over the 5 days, with 2 sessions most days, with a lunch break in between sessions. Each session lasts between 45-90 minutes. |
|  |  |
| **9. Tailoring** | **If the intervention was planned to be personalised, titrated or adapted, then describe what, why, when and how.** |
|  | The intervention follows a standardised process of education, movement retraining and self-management planning. However, the specific contents of each section is personalised to the needs of the patient. |
|  |  |
| **10. Modifications** | **If the intervention was modified during the course of the study, describe the changes (what, why, when, and how).** |
|  | Not applicable. |
|  |  |
| **11. How well: Planned** | **If intervention adherence or fidelity was assessed, describe how and by whom, and if any strategies were used to maintain or improve fidelity, describe them.** |
|  | Not applicable |
|  |  |
| **12. How well: Actual** | **If intervention adherence or fidelity was assessed, describe the extent to which the intervention was delivered as planned.** |
|  | Not applicable. |

**References**

1. Hoffmann TC, Glasziou PP, Boutron I, Milne R, Perera R, Moher D, et al. Better reporting of interventions: template for intervention description and replication (TIDieR) checklist and guide. BMJ. 2014;348.
2. Nielsen G, Buszewicz M, Stevenson F, Hunter R, Holt K, Dudziec M, et al. Randomised feasibility study of physiotherapy for patients with functional motor symptoms. J Neurol Neurosurg Psychiatry. 2017;88:484–90. doi:10.1136/jnnp-2016-314408.
3. Nielsen G, Stone J, Matthews A, Brown M, Sparkes C, Farmer R, et al. Physiotherapy for functional motor disorders: a consensus recommendation. J Neurol Neurosurg Psychiatry. 2015;86:1113–9. doi:10.1136/jnnp-2014-309255.
4. Edwards MJ, Fotopoulou A, Parees I. Neurobiology of functional (psychogenic) movement disorders. Curr Opin Neurol. 2013;26:442–7. doi:10.1097/WCO.0b013e3283633953.
5. Nielsen G, Ricciardi L, Demartini B, Hunter R, Joyce E, Edwards MJ. Outcomes of a 5-day physiotherapy programme for functional (psychogenic) motor disorders. J Neurol. 2015;262:674–81. doi:10.1007/s00415-014-7631-1.
